# Gene Therapy for the Treatment of Parkinson’s Disease: The Nature of the Biologics Expands the Future Indications

**DOI:** 10.3390/ph5060553

**Published:** 2012-06-04

**Authors:** Massimo S. Fiandaca, Krystof S. Bankiewicz, Howard J. Federoff

**Affiliations:** 1 Translational NeuroTherapy Center, Department of Neurological Surgery, University of California San Francisco, 1855 Folsom Street, Mission Center Building, San Francisco, CA 94103, USA; Email: Krystof.Bankiewicz@ucsf.edu (K.S.B.); 2 Departments of Neurology and Neuroscience, Georgetown University Medical Center, 4000 Reservoir Road, Washington, DC 20007, USA; Email: hjf8@georgetown.edu (H.J.F.)

**Keywords:** convection-enhanced delivery, enzyme-replacement therapy, gene therapy, neurotrophic factor, Parkinson’s disease, viral vector

## Abstract

The pharmaceutical industry’s development of therapeutic medications for the treatment of Parkinson’s disease (PD) endures, as a result of the continuing need for better agents, and the increased clinical demand due to the aging population. Each new drug offers advantages and disadvantages to patients when compared to other medical offerings or surgical options. Deep brain stimulation (DBS) has become a standard surgical remedy for the effective treatment of select patients with PD, for whom most drug regimens have failed or become refractory. Similar to DBS as a surgical option, gene therapy for the treatment of PD is evolving as a future option. In the four different PD gene therapy approaches that have reached clinical trials investigators have documented an excellent safety profile associated with the stereotactic delivery, viral vectors and doses utilized, and transgenes expressed. In this article, we review the clinically relevant gene therapy strategies for the treatment of PD, concentrating on the published preclinical and clinical results, and the likely mechanisms involved. Based on these presentations, we advance an analysis of how the nature of the gene therapy used may eventually expand the scope and utility for the management of PD.

## 1. Introduction

With an ever-increasing number of patients suffering with Parkinson’s disease (PD) worldwide, as the average age of the population rises [1,2], the cost of medical and pharmaceutical treatments for these afflicted individuals is climbing rapidly [3,4,5,6,7,8]. A recent estimate of the annual economic impact associated with PD in the United States (US) alone approaches $11 billion, with over half of the expense related to direct medical costs [9]. With the number of individuals suffering from PD in the US today expected to double by 2030 [10], cost-effective approaches to treatment and possible disease-modifying therapies are being aggressively pursued.

Over the last decade, gene therapy for the treatment of PD has offered promise as an effective treatment option in the face of later stages of the disease as well as providing a potential disease-modifying alternative. With human trials using this technology progressing into later clinical phases, it is important to understand the preclinical foundations supporting this therapeutic modality, while discussing future implications to the overall treatment of PD. In this special issue of *Pharmaceuticals* dedicated to Gene Therapy, this review is intended to provide a basis for understanding the use of various gene therapy strategies for the treatment of PD. We will conclude the presentation by considering how the nature of the biologic used in PD gene therapy may ultimately expand the scope and utility of the treatment armamentarium for this neurological disorder.

## 2. A Neurotherapeutic Framework for PD

For most clinicians, the causes of PD are thought to include both environmental and genetic factors [2,11]. Except for certain rare familial cases, however, where gene alterations can be clearly defined, the specific etiology of PD in most individuals remains elusive. With the etiopathogenesis of PD being unknown in most of the afflicted patients, therefore, treatment regimens to date have been primarily relegated to the management of clinical symptoms and signs, once present. Pharmaceutical agents and neurosurgical options for the treatment of PD have been formulated to ameliorate functionality through direct effects on specific brain neurotransmitters and pathways. PD gene therapy strategies to date have been established to replace missing substrates/enzymes in defective circuitry and to support and possibly rescue degenerating nigrostriatal dopaminergic neurons.

### 2.1. Pathophysiology

**Figure 1:**
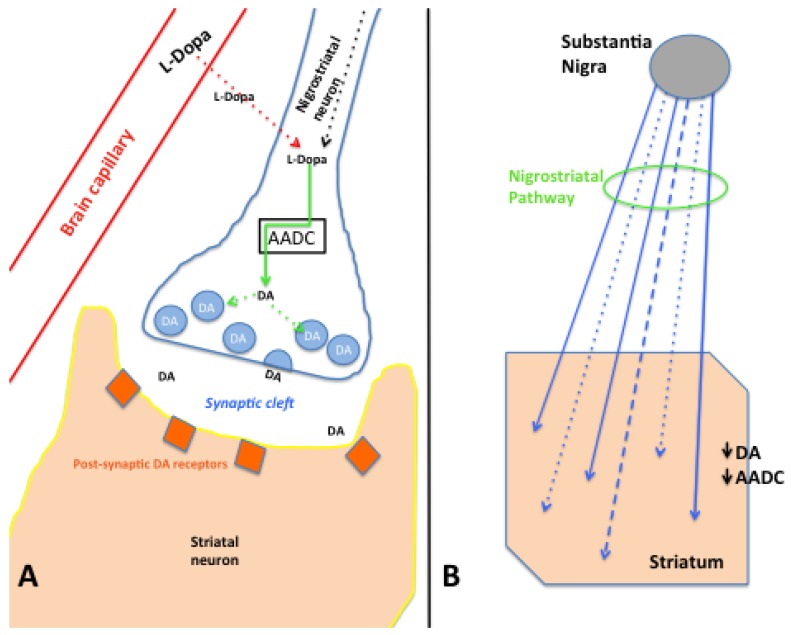
(**A**) Schematic representation of a brain dopaminergic perisynaptic region. A local capillary allows blood supply to deliver L-dopa to the brain parenchyma, traversing the blood-brain barrier and being taken up by the presynaptic dopaminergic nigrostriatal neuron (dotted red arrow). These neurons also produce L-dopa within the cell and transport it to the presynaptic terminals (dotted black arrow). At the neuron terminal, AADC (black box) converts L-dopa to dopamine (DA) (solid green arrow), allowing DA to be packaged (dotted green arrows) into presynaptic vesicles (light blue circles) for release into the synaptic cleft as part of neurotransmission. Released DA is able to interact with postsynaptic receptors on striatal neurons; (**B**) Schematic of the degeneration of the nigrostriatal pathway. Nigrostriatal dopaminergic neurons (blue arrows) originate primarily within the midbrain substantia nigra (dark gray oval) and extend into the striatum. With progressive degeneration of the nigrostriatal pathway (green circle), fibers lose their normal caliber and eventually lose function (dashed blue line) and degenerate (dotted blue lines), in association with loss of dopaminergic perikarya in the substantia nigra. Striatal biochemical assays show decreased DA and AADC levels associated with nigrostriatal degeneration.

In normal brains, dopamine (DA) is produced within the nigrostriatal neurons via enzymatic conversion of intrinsically produced (or derived from blood-borne pharmaceuticals) L-dopa by aromatic L-amino acid decarboxylase (AADC) within the nerve terminals (Figure 1A). DA, manufactured, stored, and released from these nigrostriatal terminals within the putamen (PUT) provides afferent neurotransmitter input to intrinsic neurons that participate in a modulatory feedback loop (that includes at least the globus pallidus (GP), subthalamic nucleus (STN), and substantia nigra (SN), regulating skeletal muscle motor activity. Working in concert with the neocortex via the thalamic, brainstem, cerebellar, and spinal cord centers, normal muscular tone is maintained and complex motor function is accomplished.

Since the first clinical description of PD by James Parkinson in 1817 [12], the clinical hallmarks of the disorder have become well known, and feature tremor, rigidity, hypokinesia, and postural instability. Neuropathologically, a progressive degeneration of the dopaminergic nigrostriatal neuronal pathway is found (Figure 1B), first documented by Lewy in 1912 [13]. Specific dopaminergic neuronal loss within the SN pars compacta (SNc) is associated with marked reductions in AADC for DA production, and thereby, the striatal levels of DA, which modulate somatic motor control. With a decline in striatal DA levels to below 20% of normal [14,15,16], clinical PD becomes manifest.

### 2.2. Medical Therapeutics for PD

From a drug delivery perspective, therapeutic agents used in the medical management of PD are typically classified as “indirect” treatment options, since their pharmacokinetics rely on systemic uptake and redistribution across the blood-brain barrier (BBB) and into the central nervous system (CNS). Effective medical management of PD dates back to the 1960s, after the introduction of L-dopa (levodopa) therapy by Cotzias and his associates [17]. Levodopa revolutionized the medical management of PD. Orally administered Sinemet^®^ (levodopa/carbidopa—carbidopa blocks the peripheral effects of levodopa and does not cross the BBB) is absorbed into the bloodstream from the gastrointestinal tract and levodopa is delivered (primarily via diffusion) across the BBB to impact CNS production of DA. Blood levels of levodopa, therefore, significantly determine brain levels of this molecule, with its rapid conversion to DA via AADC (rate-limiting enzyme, with respect to levodopa) within nigrostriatal neurons.

For the first decade of levodopa therapy, individual patients experience excellent resolution of their clinical manifestations of PD, without noticeable side effects. As the disease progresses, however, the nigrostriatal neuronal degeneration leads to continuing loss of the AADC enzyme activity, requiring increased doses of the Sinemet^®^ for therapeutic efficacy. With further progression of the disease, and higher doses of L-dopa required to drive DA production via the reduced levels of AADC, patients begin to suffer significant motor fluctuations (on/off phenomena) and disabling dyskinesias [18]. Due to these levodopa-associated side effects, alternative therapeutic options have been developed, including DA agonists, amantadine, anticholinergics, selegiline and others [19]. Despite the myriad of medical options available for the management of PD, none have provided a safe, long-lasting clinical benefit or offered to retard or arrest the progression of the disease [20].

### 2.3. Surgical Therapeutics for PD

Differing from medical therapeutics, neurosurgical treatments for PD are considered “direct” treatment options, with therapeutic interventions being directly within the CNS. Neurosurgical approaches to the brain allow the BBB to be bypassed and focal treatments rendered directly to the abnormal anatomical and/or physiological loci. Although the gross anatomy of the basal ganglia had been well described and illustrated by Willis and Wren as early as 1664 [21], their histological and physiological characteristics did not come to light until the 20th century [22]. With an improved understanding of the anatomy and physiology associated with PD [23,24], neurosurgeons of the early 1900s innovated many surgical remedies, often based on animal studies, to regulate abnormal motor control and tone via lesioning of the cortical and subcortical brain structures [25,26]. Until recently, neurosurgical treatment options for PD [22] had their greatest impact on the management of afflicted individuals prior to the discovery and widespread use of levodopa and other medical formulations. Even more unique neurosurgical treatments for PD were advanced through significant investigations over the last 30 years, in an attempt to replete the degenerating nigrostriatal neurons in afflicted patients using either fetal neurons [27,28,29,30] or adrenal medullary neurons [31,32]. The lack of success with these cell/tissue implantation studies, eventually led to preclinical studies that questioned the need of viable implanted neurons for a beneficial effect [33,34,35,36,37]. With the resounding answer to these investigations being that neuronal cells were not necessary for a therapeutic effect in experimental models of PD, the basis for neurotrophic factor (NTF) treatments and gene therapy were established.

In parallel, technological advances in brain imaging and stereotactically implantable, self-contained, deep-brain stimulation (DBS) systems, have allowed a renaissance in neurosurgical treatment options for PD [38,39]. Additional direct pharmaceutical delivery strategies for the treatment of PD continue to include the implantation of engineered cell lines [40], encapsulated tissue/cells [41,42], and direct pharmaceutical infusions of growth factors [43,44,45,46,47], all which provide trophic influence on neurons of the brain parenchyma.

### 2.4. Insights from Preclinical Gene Therapy Studies for PD

The most relevant insights associated with preclinical gene therapy studies for PD are primarily concerned with: (1) the preferred vector for gene delivery; (2) the optimal delivery platform for bypassing the BBB; and, (3) how to optimize gene delivery within the target. Expansion of specific topics related to the therapeutics used in gene therapy will be covered in later sections of this manuscript.

There are three major reasons why adeno-associated virus, serotype 2 (AAV2) vector has achieved a preferred status in gene therapy studies within the brain. First, AAV2 vector is highly neuron-specific [48,49], and has low affinity for transducing antigen-presenting cells (APCs) in the brain [50]. While the AAV2 vector can provoke, under some circumstances, a mild humoral response, especially when the vector escapes from the brain into the peripheral circulation, the immune response is typically directed against capsid proteins rather than the encoded transgene. The issues concerning AAV immunology has been reviewed much more thoroughly elsewhere and the interested reader is directed to more in-depth discussions [51,52]. The second advantage of the AAV2 vector is historical. There is considerably more experience with the clinical use and safety data with AAV2 vector compared to any other AAV vector serotype, and this enhances the perceived safety of this vector, especially for neurological studies. The third factor is that the vector has been manufactured at industrial scale for some years by commercial entities [53]. Such common biomanufacturing capabilities suggest that even clinical protocols sponsored by academic groups can expect to transition expeditiously into commercial development via these biopharmaceutical companies, once safety and efficacy is documented.

The optimal direct delivery platform for therapeutics into the CNS remains a topic of debate, but from our perspective, convection-enhanced delivery (CED) [54,55,56,57] offers significant advantages over diffusion-based delivery methods, especially when CED is optimized with real-time imaging [58,59]. Direct non-convective intraparenchymal injections and implanted eluting polymers depend on diffusion to deliver their therapeutic agent to the surrounding brain parenchyma, with an achieved diffusion limit of several millimeters [60,61], thereby significantly limiting the treatment volume. CED has consistently delivered higher doses of therapeutics over a more significant clinical volume of distribution within CNS parenchyma [62,63,64,65].

Optimizing gene delivery within the CNS target includes the use of a target specific vector with high transduction efficiency, such as AAV2 for neuronal populations [48,49], optimizing delivery cannula placement within the target [66,67], the use of a reflux-resistant cannula [68], the use of real-time imaging [49,59,64,69,70,71,72,73,74,75,76,77,78,79,80,81,82], and optimizing the tissue affinity of the infusate [83] and vector [57]. Recently, we have also utilized thalamic targets and their cortical projection fibers to transduce specific cortical regions with gene therapy, via anterogradely-transported viral vectors [84]. Although retrograde transmission to the nigra from striatal targets has been suggested as a mechanism of action for some NTF gene therapy, this mechanism of action remains controversial in both preclinical and clinical trials [85].

### 2.5. Insights from Clinical Gene Therapy Trials for PD

Since 2003, there have been five major research groups worldwide participating in clinical trials for the treatment of PD utilizing gene therapy [85,86]. So far, clinical investigations have advanced through the Phase II trial process without evidence of major safety concerns utilizing both the AAV2 vector (used by the three clinical groups in the US, and a group in Japan) and a lentivirus (LV) vector (in European Union (EU)-based studies). While a few complications were associated with the stereotactic surgical procedures (hemorrhages, venous infarcts) [87], none were directly attributed to the viral vectors or transgenes. Hemorrhagic complications of functional neurosurgery are well known, infrequent, and have been markedly reduced through the use of image-guided approaches [88].

**Figure 2:**
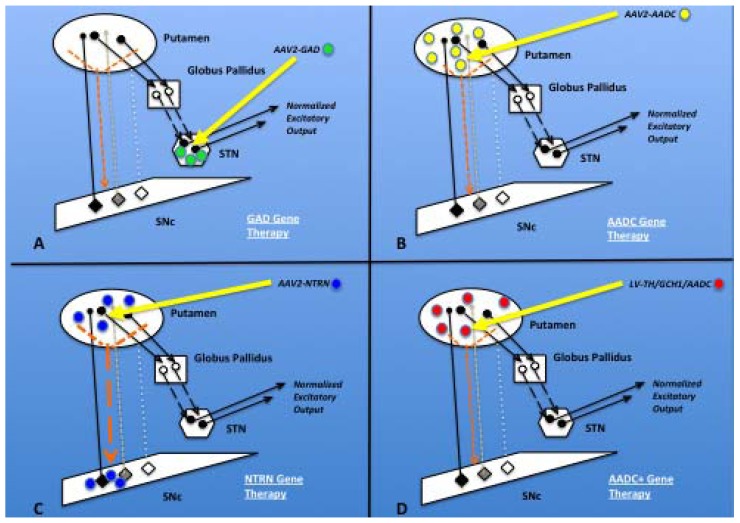
Current gene therapy treatments for PD. In each panel (**A–D**), the nigrostriatal (NS) neuron cell bodies (diamond shapes) are shown within the substantia nigra pars compacta (SNc), with normal (black), degenerating (gray), and degenerated (white) perikarya and axons projecting to the putamen. The reciprocal striatonigral pathway is depicted (orange dashed arrow). Typically, NS dopaminergic (DAergic) neurons (black diamond) influence putamenal neurons. Excitatory putamenal neurons (black circles and arrows) project to the globus pallidus, where inhibitory neurons (white circles and arrows) are activated. Inhibitory tone from the globus pallidus suppresses excitatory output from the subthalamic nucleus (STN), and thereby maintains normal motor control. In PD, the loss of excitatory DAergic input to the putamen, therefore leads to a relative increase output from the STN, leading to motor dysfunction. (**A**) GAD gene therapy. Stereotactic delivery (yellow arrow) of AAV2-GAD (green circle) into the bilateral STN allows increased local GABA production, thereby inhibiting excitatory STN output and improving motor system dysfunction; (**B**) AADC gene therapy. AAV2-AADC (yellow circle) delivery to the bilateral putamen replaces lost AADC. Re-establishing the DAergic stimulation of striatal neurons helps normalize the motor feedback loop through globus pallidus and STN; (**C**) NTRN gene therapy. AAV2-NTRN (and GDNF) (blue circle) delivery provides local production of NTRN and enhances residual DAergic cell function within the putamen. Retrograde transport of AAV2-NTRN within residual intact DAergic nigral neurons, as well as anterograde transport of AAV2-NTRN via the striatonigral pathway (dashed orange arrow) to the SNc, further improves DAergic neuronal function and survival. Injection of vector into SN is not shown but would increase the amount of AAV2-NTRN within the SN, independent of the anterograde transport pathway; (**D**) AADC+ gene therapy. Lentiviral (LV) vector delivery, carrying the tripartite gene construct for the dopamine biosynthetic enzymes (TH/GCH1/AADC) (red circle), allows restoration of excitatory DAergic tone within the putamen.

The five research groups, involved in clinical gene therapy studies for PD, have investigated four different transgene constructs to date (Figure 2). Four of the groups have used gene therapy for enzyme replacement, while a fifth group delivered a neuronotrophic factor gene. A US team successfully delivered the glutamic acid dehydroxylase (GAD) gene (enzyme replacement) with an AAV2 vector to the STN of PD patients in both Phase I and II trials, and has shown significant efficacy in peer-reviewed publications [89,90], prompting discussions of proceeding to a more definitive Phase III trial. With this treatment paradigm, the delivered transgene produces an inhibitory neurotransmitter (gamma (γ) amino butyric acid, GABA) within the STN; suppressing abnormal firing of the STN neuronal population associated with altered motor function in PD. Since the corporate sponsor for this approach, Neurologix Inc. (NRGX), recently entered Chapter 7 proceedings [91], a Phase III trial appears unlikely. Also utilizing the AAV2 vector, a second US group and a group from Japan delivered the AADC gene (enzyme replacement) to the bilateral PUT of PD patients in Phase I trials [86,92]. The AADC transgene within neurons of the PUT allows replacement of the lost intrinsic nigrostriatal AADC seen with PD, and allows restoration of local DA production, particularly with the pharmacologically provided substrate levodopa. Data from the AADC trials disclosed robust evidence of transgene expression via postoperative positron emission tomography with 6-[^18^F]-fluoro-L-*m*-tyrosine (FMT-PET) within treated PUT, as well as a suggestion of clinical improvement [86,87], despite the possibility of less than optimal targeting of the infusate [93].

Follow-up nonhuman primate (NHP) studies by the US AADC investigators have indicated the importance cannula positioning within the target PUT [67], as well as the need for increasing the infusion volume per hemisphere [59] to optimize target coverage. The third group of US investigators has completed Phase I and II clinical trials utilizing the AAV2 vector for neurturin (NTRN) gene therapy (neuronotrophic factor replacement) within the PUT of PD patients [94,95]. Clinical efficacy was not shown in the Phase II trial of NTRN gene therapy [94]. Investigators found no evidence of improvement in PET signal within treated PUT, and autopsy evaluation of implant sites were suggestive of less than optimal target coverage by the transgene [96]. The lack of clinical efficacy in the NTRN Phase II trial [94], in addition to further preclinical investigations [97], has prompted this research team to target not only the PUT but also the SN in a follow-up Phase II investigation (ClinicalTrials.gov identifier NCT00985517), scheduled for completion in late 2014. Finally, a fifth group of investigators, from the EU, utilized the larger LV vector to deliver three therapeutic genes (enzyme replacement) from the DA synthesis pathway (including AADC) to treat PD patients in Phase I/II trials [98] (ClinicalTrials.gov identifier NCT00627588), due for completion in 2012. According to preliminary unpublished reports [99], safety and prolonged clinical efficacy has been shown in the treated PD patients. In considering the information developed by four research/clinical groups described above, as well as other investigators in the field, we support of the following steps moving forward in additional Phase II or Phase III clinical trials utilizing gene therapy for the treatment of PD:

1. The use of optimized CED techniques with real-time imaging and stereotactic guidance to confirm the targeting and distribution of the infusate, especially in large targets, such as PUT. There is no supportive evidence for using non-convective delivery methods (hand injection) for gene therapy within the CNS, even within a minute target such as STN. Postmortem assessments of targeted PUT show that non-convective delivery methods offer limited transduced volumes of distribution within large brain targets. Real-time image-guidance is necessary to not only improve safety, but also to confirm target acquisition and coverage.
2. The importance in using a specifically designed, MRI-compatible, CED infusion cannula to minimize brain trauma, while optimizing reflux-free convection of therapeutics within the brain.
3. Increasing the infusion volume for putamenal infusions in an attempt to cover and transduce >50% of the post-commissural putamenal volume.
4. Since the vectors used (AAV2 and LV) and transgenes appear safe at increasing titers, we support using the higher vector titer found to be safe based on preclinical and Phase I studies.

It is obvious to us that there is room for improvement in utilizing gene therapy for the treatment of PD going forward. The following topics further focus on the therapeutic options with gene therapy in PD, and how they may be implemented in the future.

### 2.6. Delivery Vectors

#### 2.6.1. AAV

The adeno-associated virus (AAV), is a member of the nonpathogenic parvovirus family, and has been used to deliver gene therapy constructs of approximately five kilobases [100], when flanked by two inverted terminal repeats (ITRs) [101]. Most of these viruses contain single-stranded DNA, with the AAV2 clone being the first clinical vector, established in 1982 [102]. Since then, more than 80 AAV gene therapy clinical trials have been initiated worldwide [103], without evidence of major immunological reactivity or direct pathogenesis. Importantly, the AAV2 serotype infects non-dividing cells and appears to be more neuronotropic than the AAV1 serotype [49], making it a better choice for targeting neurons and not the surrounding glia within the brain.

In humans, wild type AAV integrates within the host genome at a specific site on chromosome 19 [104,105], with other genome incorporations occurring at a negligible rate. Most of clinical gene therapy vectors used to date have been recombinant AAV2 (rAAV2) vectors, and have been used to transduce brain, muscle, liver, retina, and lungs, often requiring several weeks for peak expression [106]. Transduction competency with all rAAVs depends upon efficient cellular binding, entry and trafficking, nuclear entry, uncoating, and second-strand synthesis. Trafficking and second-strand synthesis are notable rate-limiting processes in AAV gene expression [107,108]. Both the intracellular phosphorylation state of protein FKBP52 and epidermal growth factor receptor kinase signaling have been implicated in the regulation of these rate-limiting steps of AAV expression [109,110,111].

Specificity and efficacy of transduction has also been associated with specific cell surface receptors. The AAV5 vector results in a widespread pattern of robust expression within the brain and spinal cord, related to sialic acid receptor binding, which it shares with certain CNS-specific infectious agents [112]. Alternatively, AAV2 enters cells primarily via the heparan sulfate proteoglycan primary receptor and fibroblast growth factor receptor 1 co-receptors [113,114]. Local population densities and affinities of these (and other) receptors are expected to affect the AAV transduction efficiencies in various CNS loci.

Recent studies have looked at the potential for other AAV serotypes (and chimeric pseudotypes) to be effectively used for gene therapy within the CNS, related to tropism differences they have with AAV2 [115,116]. AAV1 and AAV5 exhibit more robust transduction frequencies than AAV2 in all CNS regions [115,117], and show the most widespread affinity to CNS parenchyma [118]. AAV4 has shown a predilection for ependyma and astrocytes [119]. Most recently, rAAV9 has received attention [118,120] for its potential to transduce astrocytes and neurons within the CNS following peripheral intravascular (iv) administration. Differential targeting of the spinal cord in adult animals (especially humans) following iv administration of rAAV9 [120], offers a potential to bypass the BBB and delivering a genetic payload without invasive surgical procedures. Obviously, further preclinical gene therapy investigations are warranted, along with requisite clinical trials, to determine the most promising AAV alternatives to AAV2 in PD and other CNS diseases.

#### 2.6.2. Lentivirus

LVs are derived from a group of pathogenic retroviruses that include HIV [121]. LVs typically contain a double-stranded RNA core along with a reverse transcriptase protein for conversion of the RNA payload to double-stranded DNA, allowing the latter to be incorporated within the infected host cell nucleus. LVs integrate into non-dividing as well as dividing cells, including stem cells [122]. Feline and equine LVs, in particular, appear to be non-pathogenic to humans [121,123,124]. The larger size of the LV vectors, compared with the AAV vectors, allows larger gene constructs (up to 9 kilobases) to be included within LV vectors for therapeutic delivery [125,126], with additional adaptations allowing improved packaging effectiveness [127,128].

LV vector pseudotyping to generate amphotrophic envelopes has allowed the development of tropism to include that of neurons and glia [129,130,131,132], from the “wild-type” that typically is preferential to mononuclear phagocytes [133,134]. Long-term neuronal transgene expression, with minimal inflammatory response is associated with LV vector use within the CNS [131]. Multicistronic gene constructs have been developed for use in PD gene therapy [135,136].

While the development of oncogenic mutations with the use of LV vectors (and with other retroviruses) continues to be a primary safety concern, no evidence for tumorigenesis has been documented in a long-term PD clinical trial using such genetic delivery vehicles [99]. Additionally, the development of non-integrating lentiviral (NIL) vectors, whose genome exists as a separate linear or circular entity within the host nucleus, may continue to provide efficacy while increasing the theoretical safety of LV vectors in future clinical trials [137,138,139].

#### 2.6.3. Other Viral or Non-Viral Gene Therapy Delivery Platforms

As of 2011, the adenovirus (AdV) vector was the most commonly used viral delivery vehicle in human clinical gene therapy trials [103], with its frequency of use peaking before 2000. AdV vectors are non-enveloped virions with a double stranded DNA genome containing 26–45 kilobases, and the capacity to carry up to 30 kilobases of exogenous genetic constructs [140]. While having a broad tissue tropism, including post-mitotic cells such as neurons, their use has been primarily peripheral to the CNS [140].

The herpes simplex virus-1 (HSV-1) has a natural tropism for the CNS [141,142,143,144] and is known to develop a lifelong presence within sensory neurons. Carrying a 152 kilobase double-stranded DNA genome, HSV-1 has a protein capsid surrounded by a lipid membrane envelope and an intervening protein tegument, and is the largest and most complex viral vector being developed for gene therapy [140]. Two major types of vector systems are based on the HSV-1 virus, and include the recombinant viral vector and the HSV-1 amplicon. The recombinant vector is generated by replacement of certain nonessential viral genes with transgene constructs; accommodating up to 40 kilobases of transgenic DNA [140]. The HSV-1 amplicon system carries a very limited viral genome, thereby increasing the carrying capacity for foreign expression cassettes [145], and reduces associated cellular toxicity and immune system activation compared to recombinant HSV-1 [146]. Clinical HSV vector use in the CNS to date has been limited to neurooncology [147], with the HSV-1 amplicon vectors not yet clinically tested. Preclinical investigations, however, have shown promise for HSV vector use as future delivery vehicles for CNS gene therapy [148,149,150,151,152].

The use of liposomal delivery for cellular transfection continues its development, and, while it features complicated methodological requirements, it offers distinct advantages over viral-mediated delivery systems [153,154]. Although AAV and lentiviruses appear to have low immunological consequences in humans, liposomes appear to be devoid of immunological effects *in vivo* [154]. Systemic liposomal delivery has been enhanced by avoiding immunological clearance through modification of surface moieties (including polyethylene glycol (PEG) or derivatives, PEGylation) and has already a track record for use with CED within the CNS [155]. Importantly, liposomes are relatively inexpensive to produce, do not impose the constraints on size of the genetic payload, as is seen with many viral vectors, and can be engineered to take advantage of surface targeting molecules for enhanced targeting specificity [154]. Disadvantages for liposomal delivery systems have included the poor cellular uptake of early constructs, but recent advances have dramatically improved efficiency of transfection using these non-viral vectors [155,156,157]. Additional disadvantages have been seen with repeated use of PEGylated liposomes for direct therapeutic brain delivery, implicated in complement activation [158], and the development of the potentially life-threatening complement activation-related psuedoallergy (CARPA) [159,160]. Important investigations continue to be required before the common clinical implementation of liposomal delivery for neurodegenerative diseases.

### 2.7. Neurotrophic Factors (NTFs)

NTFs are secreted proteins that play a major role in the growth and development, maintenance, and plasticity of the CNS [161]. At least four major families of molecules make up the NTF proteins: (i) the neurotrophin family [162,163,164,165,166]; (ii) the glial cell line-derived neurotrophic factor (GDNF) family of ligands (GFLs) [161,164,165,166,167,168]; (iii) the neurotrophic cytokines (neurokines) [166]; and (iv) the recently described family that includes the cerebral dopamine neurotrophic factor (CDNF) and mesencephalic astrocyte-derived neurotrophic factor (MANF) [161,169].

To focus our discussion to NTFs that are currently utilized for clinical gene therapy trials for PD, we limit our discourse to two members of the GFLs, NTRN and GDNF. We acknowledge that future investigations may find merit and utility for some of the other NTFs referenced in the previous paragraph, and/or those yet to be discovered.

GFLs are a subfamily within the transforming growth factor beta (TGF-β) superfamily [170,171,172]. GDNF, the first member of the GFLs was purified in 1993, and noted to promote survival and morphologic differentiation of dissociated embryonic midbrain dopaminergic neurons, while increasing their high affinity DA uptake [173]. Sequence analysis implies that GDNF is synthesized as a precursor molecule that undergoes processing and is finally secreted as a 134 amino acid protein [170,173]. GDNF, NTRN, artemin (ARTM), and persephin (PSPN) comprise the GFL family. An additional three years of investigation, following GDNF’s discovery, resulted in the identification and purification of NTRN [174]. The mature NTRN molecule is secreted as a 100 amino acid protein, sharing 42% sequence similarity with GDNF [174]. The ARTM and PSPN act only outside the brain and spinal cord, since they lack specific co-receptors within the CNS [161].

The signaling pathway for GFLs is unique among the NTFs, and was described by several groups in 1996 [175,176,177,178,179]. Dimeric GFLs interact with two molecules of the GDNF family receptor α (GFRα 1–4) co-receptor, making a conjugated ligand-receptor complex [161,170]. GFRα ligation with a GFL results in engagement with tyrosine kinase Ret (Rearranged during transfection [180]) receptor tyrosine kinase (rRTK) [176,181,182,183]. At physiologic levels, GDNF and NTRN bind preferentially to the GFRα1 and GFRα2 co-receptors, respectively [176,178,184]. Supraphysiologic levels of both of these GFLs (attained with infusions or gene therapy) lead to binding to either GFRα1 or GFRα2 co-receptors [185]. GDNF and NTRN are transcribed in the striatum, while GFRα1 and Ret are expressed in nigrostriatal neurons of the SN [184,186,187,188,189,190,191,192]. Since SN DA neurons express GFRα1 and not GFRα2 [165], both GDNF and NTRN act through the GFRα1 co-receptor on dopaminergic nigrostriatal pathways. With an intact nigrostriatal pathway, co-receptor bound GDNF (or NTRN) is retrogradely transported to the SN pars compacta (SNc) DA perikarya to interact with the local transmembrane rRTK [176,181,182,183]. This conjugated GFL-receptor complex then binds and activates rRTK, which is the common signaling receptor for all four GFLs [161], activating diverse signaling cascades shown to promote cellular survival and appropriate responses to oxidative stress. Additionally, rRTK-independent signaling of GFLs has been associated with neurite outgrowth and synaptogenesis [161].

#### 2.7.1. Neurturin (NTRN)

As a result of patent rights issues for GDNF protein and the GDNF gene [193], NTRN gene therapy was the first GFL-gene therapy to be commercially developed (Ceregene Inc., San Diego, CA, USA) in combination with an AAV delivery vector (CERE-120, AAV2-NTRN) [194]. As previously noted, NTRN shares significant functional characteristics with GDNF [195,196]. NTRN has shown similar neuroprotection and neuroregeneration profiles as GDNF to chemical lesioning of the nigrostriatal pathway in rats [197] and NHPs [198,199,200]. Preclinical investigations in young and aged NHPs [201,202] indicate that Ret expression in the target nigrostriatal pathways does not change significantly with age, and that stereotactically-delivered CERE-120 is safe at dose multiples of up to 100 times greater than required for *in vitro* efficacy. In a follow-up placebo-controlled NHP study using CERE-120 in a model of acute methylphenylpyridine (MPTP) toxicity [197], significant behavioral improvement (compared to controls), similar to that seen with GDNF in another NHP study [200], was noted at 4 months following gene therapy, and remained stable for up to 10 months. In each NHP, the investigators targeted the caudate, PUT, and SN with either CERE-120, or control solutions [197]. The authors described that CERE-120 animals displayed significant preservation of dopaminergic nigral perikarya, increased levels of growth factor-responsive nuclear and cytoplasmic proteins in nigral dopaminergic neurons, and partial maintenance of striatal dopaminergic innervation [197].

Using this preclinical data as validation, investigators initiated a Phase I human clinical trial with CERE-120 in PD patients (ClinicalTrials.gov Identifier: NCT00252850) [95], which confirmed the safety of the stereotactic procedure and the CERE-120 dose delivered within the PUT of PD patients. Disappointingly, the subsequent Phase II study of intraputaminal CERE-120 (ClinicalTrials.gov Identifier: NCT00400634) failed to show significant efficacy compared to a sham surgery control group at 12 months following treatment [94]. Neuropathological assessment of a patient that succumbed to an unrelated malady, and showed no significant improvement in his PD following CERE-120 gene therapy, featured minimal NTRN expression at the sites of striatal infusion or within the SN [96]. These latter pathological results have raised the issue of possible altered transport of NTRN from PUT to SN in PD, prompting a proposed CERE-120 trial (ClinicalTrials.gov Identifier: NCT00985517) where both the PUT and SN will be directly targeted, in an effort to improve NTRN efficacy on the degenerating nigrostriatal pathway, as had been shown in a NHP preclinical study [197].

#### 2.7.2. GDNF

The source of striatal GDNF appears to be from a population of parvalbumin-positive interneurons, making up <1% of all striatal neurons [203]. GDNF messenger RNA (mRNA) has been found to be markedly upregulated in the PUT of PD patients with significant loss of SNc neurons [204]. Additionally, brain tissue of PD patients has shown reduced GDNF and GFRα1 levels [191,205], in some studies, as well as stable rRTK and GFRα1 levels in others [204,205]. Evidence exists for increased protein phosphorylation via GDNF-GFRα1-rRTK binding/activation that leads to increase DA biosynthetic capability in nigrostriatal projections, as well as DA efferents to the SN pars reticulata (SNr) that modulate motor function via thalamocortical projections [206]. Despite the investigations mentioned above, and many others, there remains an incomplete understanding of the specific mechanisms of action for GDNF protein on the nigrostriatal and other brain pathways. The potent trophic effects of GDNF on nigrostriatal DA neurons have led to clinical investigations using both recombinant protein infusions and gene therapy [207,208], building on our understanding of GDNF transport in the CNS [209].

Mixed results have been associated with clinical GDNF infusions into the CNS of PD patients [43,44,45,47,210,211]. Because of the variable efficacy noted, and concerns for potential treatment-associated neuropathology [212], Amgen (Amgen Inc., Thousand Oaks, CA, USA), which controlled the patents for the GDNF protein and the GDNF gene, elected to cease all GDNF infusion trials in 2004 [213,214,215]. In 2008, after extensive negotiations, Amsterdam Molecular Therapeutics (AMT, Amsterdam, The Netherlands) finally obtained a license from Amgen to advance GDNF gene therapy for PD [216]. At this time there is optimism that a Phase I PD gene therapy trial utilizing GDNF will move forward [217], especially with the recent FDA approval of the IND (IND 14996) [218].

With GFL gene therapy, the specific anatomical targeting requirements remain controversial, especially after recent Phase II results with NTRN. A major controversy associated with using GDNF and NTRN in future PD clinical trials has to do with where these trophic factors should be directly targeted. Despite low levels, maintenance of dopaminergic fibers and synapses require the presence of GDNF in adult striatum [219,220]. Targeting of the PUT for GFL gene therapy has been the default option, based on early experience in rodent studies suggestive of retrograde transport of GDNF in the nigrostriatal pathway [221]. Striatal injections of AdV-GDNF protected nigrostriatal terminals (or stimulated neuronal sprouting) and nigral cell bodies, while maintaining behavioral function in a rodent model of PD, while SN injections did not [222]. Likewise, similar preclinical experience with GDNF and NTRN gene therapy in NHPs was noted utilizing striatal targets [197,198,200,223,224,225]. Lack of efficacy in the Phase II CERE-120 study [94], despite the predicted results from the mentioned preclinical investigations for both GDNF and NTRN, raise questions regarding: the appropriateness of the animal models used [226]; possible abnormal retrograde transport to SN due to degeneration of the nigrostriatal pathway in PD [94]; and/or, a lack of significant target coverage within the putamen [97].

With the MPTP parkinsonian NHP model [227,228], there is a 4-week period after acute dosing during which nigrostriatal degeneration occurs [229]. GFL-gene therapy before or during this time interval, may not optimally illustrate the restorative nigrostriatal responses needed in PD, but may better depict neuroprotection from a toxin [226]. Using an NHP parkinsonian model with GDNF (or NTRN) gene therapy, well beyond the development of a stable nigrostriatal lesion, may better mimic the clinicopathologic conditions seen in PD [226].

AAV2 vectors have been noted to distribute via anterograde (and not retrograde) transport [84,230]. Recent studies using AAV2-GDNF delivery to thalamus [84] and PUT [226,231,232], have indicated that vector transport and GDNF expression occurs at sites of efferent projections from the infusion site. In unilateral 6-hydroxydopamine (6-OHDA)-lesioned rats, striatal delivery of AAV2-GDNF showed bilateral GDNF immunoreactivity (IR) in GP, STN, and SN (SNr and SNc), except on the lesioned side, where 6-OHDA eliminated SNc cell bodies [232]. Injection of AAV2-GDNF into the SN (covering both SNc and SNr regions) resulted in striatal distribution only in the unlesioned hemisphere, and no distribution was noted in GP or midbrain [232].The distribution of AAV2-GDNF from PUT to GP, STN, and SN is identical in aged, non-MPTP-treated [231] and young, MPTP-treated [226] NHPs; suggesting that this delivery is independent of the integrity of the nigrostriatal DA pathways. Distribution of AAV2-GDNF after SN infusion in NHP has been more widespread, localizing to mesencephalic and diencephalic structures, as well as cortical regions [233]. Significant weight loss has been seen in humans receiving GDNF via intraventricular infusions [43,44] and with SN GDNF-gene therapy infusions in both rodents and NHPs [231,234]. While AAV2-GDNF gene therapy into PUT of aged [235] and parkinsonian [236] NHPs has been associated with increased FMT-PET uptake, intense GDNF fiber and extracellular IR, increased DA turnover, and enhanced locomotor activity, direct infusion of viral vector-GDNF to SN has not been as effective as when delivered into PUT in both aged and parkinsonian animals [222,231]. The data is most supportive, therefore, of striatal delivery of AAV2-GDNF in future trials, rather than within the SN.

Volumetric transduction for a large structure such as PUT is more difficult than for a smaller target, such as STN. While target coverage may be important for clinical efficacy in PD gene therapy trials, it has been difficult to document. *In vivo* MRI analysis for an AADC gene therapy trial [93] and postmortem histological assessments for a CERE-120 study [97] have estimated up to 15–25% coverage of post-commissural PUT volume with therapeutic transgene. Although optimal targeting parameters for CED into NHP putamen, thalamus, and brainstem have been described [66,67], and translation of this preclinical data for human trials has been attempted [237], use of this information in the clinical setting remains problematic. Although volumetric coverage of the target assessed with real-time convective delivery (RCD) correlates well with transgene expression for non-secreted proteins such as AADC [49], such a close relationship may not exist for secreted reporter proteins, such as GDNF (or NTRN) due to diffusion or transport out of the original target. Future clinical trials will have the opportunity to use RCD [58,59] to determine the extent of target coverage, and attempt to correlate it with clinical efficacy, despite some remaining limitations.

### 2.8. Enzyme Delivery/Replacement

Although three different gene therapy enzyme delivery/replacement strategies have been utilized so far to treat PD in the clinic, the future for this therapeutic option is only limited by our understanding of the pertinent physiological/anatomical disruptions in the disease.

#### 2.8.1. GAD

L-Glutamic acid (glutamate) decarboxylase (GAD; EC 4.1.1.15) is the rate-limiting enzyme for the synthesis of GABA from glutamic acid in the mammalian CNS. GABA is a major inhibitory neurotransmitter in both vertebrate and invertebrate systems [238]. Two GAD enzymatic isoforms (GAD65 and GAD67) have been identified [239], which differ in their overall purpose [240], as well as gene loci, subcellular localization, function, regulatory properties, and co-factor interactions [241]. GAD65 is the isoform concentrated in the nerve terminals, producing GABA for neurotransmission [242], whereas GAD67 is located diffusely within the cell and associated with various non-neurotransmitter functions [243,244,245].

While PD has been associated with disrupted levels of GABA neurotransmission [246], the cardinal symptoms and signs (see Section 2.1) result from disinhibition of the striatum associated with DA depletion due to the nigrostriatal degeneration. Loss of inhibitory DA tone on the striatum leads to relative hyperactivity of the STN output to the SNr [247,248,249,250], with resultant alteration in motor tone and function. STN ablation [251,252], electrical blockade [253,254], and pharmacological suppression [255], have all been utilized to ameliorate PD motor function. This correlation of anatomy and pathophysiology led to the development of GAD gene therapy within the STN for GABA production and suppression of STN excitatory efferents to SNr in an animal model of PD [256], and eventual translation to a clinical trial [89], which featured delivery of both GAD isoforms in a 1:1 ratio. As described previously in this manuscript (see Section 2.5), clinical outcomes with this form of gene therapy have been safe and efficacious through Phase II.

#### 2.8.2. AADC

Aromatic L-amino acid decarboxylase (AADC; EC 4.1.1.28) has a genetic locus found on human chromosome 7 [257] and is a rate limiting enzyme in DA production when levodopa is used to treat PD. In the latter circumstance, oral doses of levodopa are directly converted to DA via AADC upon the drug’s entry into the brain from the blood [258]. The loss of therapeutic efficacy of levodopa in later stages of PD [259,260] is associated with significant drug-induced side effects [261,262] and a direct result of loss of this enzyme activity within the striatum. The replenishment of AADC within the striatum of parkinsonian rodents [263] and NHPs [50] via gene therapy has been associated with improved DA production and behavioral enhancement, with AADC gene expression observed for over 7 years [264]. AADC gene expression can be monitored via FMT-PET [265,266], and higher AAV2-AADC vector doses have been correlated with increases in striatal PET uptake and improved behavioral responses in parkinsonian NHPs [267]. These preclinical strategies have been advanced into Phase I PD trials [86,87,92] that featured safety, the suggestion of dose-dependent FMT-PET uptake, and clinical improvement. A new AAV2-AADC bridging study, utilizing MRI-guided delivery in PD patients, is scheduled to move forward by 2013 (K.S. Bankiewicz, personal communication) [268].

#### 2.8.3. TH/GCH1/AADC

TH (tyrosine hydroxylase; EC 1.14.16.2), GCH1 (guanine-5'-triphosphate (GTP) cyclohydrolase 1; EC 3.5.4.16) and AADC are necessary enzymatic components of the normal DA synthetic pathway from tyrosine [269]. The conversion of tyrosine to levodopa requires the rate-limiting enzyme TH and an essential cofactor, tetrahydrobiopterin (BH_4_), which is produced from GTP with the aid of GCH1. Production of BH_4_ by GCH1 is thought to, therefore, indirectly regulate DA production from tyrosine [270,271].

With the degeneration of the DA nigrostriatal pathway, altered levels of these synthetic enzymes and cofactors are noted in PD [272]. Besides the use of AADC alone, as previously discussed, investigators have attempted to replace various single and multiple enzymes in this DA synthetic pathway using diverse gene therapy strategies in various parkinsonian animal models. Using TH alone [273,274,275,276,277], only partial neurochemical and functional deficits were restored. Further neurochemical improvement was noted in a parkinsonian rodent model by combining TH and GCH [278,279], although behavior was not ameliorated. Finally, the use of triple AAV transduction of TH, AADC, and GCH in a parkinsonian rodent model confirmed improved DA production in association with at least a 12-month behavioral improvement following intrastriatal injection [280]. Further modification of this multiple enzyme delivery system resulted in selection of a LV vector that packaged and delivered a multicistronic (TH, AADC, and MCH1) transcription unit that showed comparable biochemical and behavioral results as to those achieved using three separate AAV vectors [135]. More recently, utilizing a similar rodent model, authors have suggested the addition of the vesicular monoamine transporter 2 gene to the previous three genes, delivered within an HSV-1 vector system, further improved neurochemical and behavioral recovery [281].

In preparation for human clinical trials, parkinsonian NHP studies using the tricistronic LV vector was able to safely restore extracellular DA and correcting motor disturbances for at least 12 months [98]. These studies have led to the previously described Phase I/II EU trial (see Section 2.5) whose published clinical results are expected within the next 12 months [99].

### 2.9. Non-Regulated *versus* Regulated NTF Gene Therapy

One of the more interesting and controversial aspects of NTF gene therapy for the treatment of PD has yet to be translated effectively from rodent studies into the parkinsonian NHP model. These forthcoming investigations will involve the efficient delivery and management of externally regulatable genes within the brain. The two best-developed *in vivo* transcriptional regulatory schemes are the tetracycline/doxycycline (TET) [282] and the rapamycin (RAPA) [283] systems, which have both been used in preclinical PD studies with AAV vectors [284,285]. Because of the packaging constraints of the AAV vector, the RAPA system requires at least two vectors to carry the complete regulated gene construct for AADC enzyme replacement [284], while the TET system for GDNF gene therapy can fit within a single AAV vector [285]. Recently, the double AAV vector for RAPA-regulated GDNF gene therapy was also effectively assessed in rodents [286]. Theoretically, such systems allow the expression or suppression of a particular gene or group of genes under the control of a promoter that is sensitive to the presence or absence of TET or RAPA.

In the majority of non-PD NHP studies conducted with these two regulatable genetic constructs, the non-primate peptides associated with the TET system elicited humoral and cellular immunological responses to peripheral inoculations, and thereby prevented long-term regulation of gene expression [287]. Conversely, RAPA uses human peptides that do not appear to cause similar immunological reactions in NHPs, and has demonstrated excellent long-term regulation of gene expression [287]. Whether brain delivery of either regulatory construct, and particularly TET, would stimulate a significant immune response needs to be critically assessed in the NHP model prior to human considerations, in addition to the possible prevention of neuronal cell death, as previously observed in rodents with RAPA [288].

Despite these advances in transcriptional regulation of gene expression in the brain, there remains a significant difference of opinion between investigators regarding the need for such regulatory control with NTF (GDNF and NTRN) gene therapy in PD [289,290,291]. The investigation of transcriptional control of NTF genes in PD appears to be warranted, as pharmacokinetic studies in rodents suggest a long half-lives for both GDNF and NTRN, following single brain infusions [292] (making it less likely that continuous transgene expression is necessary). In addition, safety considerations from both preclinical [212] and clinical [47,293] studies of prolonged, high-concentration, GDNF protein exposure have been raised. While parenchymal or intraventricular GDNF protein infusions can be terminated, or could be temporally adjusted (with implantable pump technologies) in patients, current clinical gene transfer does not offer this control of transgene expression.

Prior to definitive NHP studies with regulated NTF gene therapy can be initiated, additional valuable information must be developed from rodent studies. Important differences between non-regulated (constitutive) AAV2-GDNF (conAAV2-GDNF) expression in striatum as opposed to RAPA-regulated AAV2-GDNF (regAAV2-GDNF) were recently described [286]. With conAAV2-GDNF, the time-course of striatal GDNF expression features increasing GDNF levels to a peak over the first 30 days after vector infusion, with peak levels maintained for at least three months. The maximum levels of striatal GDNF expression with the conAAV2-GDNF vector are dose-dependent. Using three serial daily intraperitoneal (ip) RAPA doses, RAPA-regAAV2-GDNF time-course indicates an increased striatal GDNF concentration lasting between 14–28 days, with a maximum level achieved at day 4, with similar temporal results in obtained with a different inducible protein [294]. Different RAPA delivery methods, including ip, oral, and via striatal CED, altered the maximum striatal level of inducible GDNF achieved, with CED providing the most efficient dosing method [286], and suggesting poor passage of RAPA across the BBB. Obviously, additional investigations evaluating the use of different viral vectors, inducible promoters, and viral titers need to be worked out. Candidate strategies would then be evaluated in NHP PD models before being translated to clinical use.

## 3. Conclusions

### 3.1. Tailoring the Biologic to the Particular PD Stage

**Figure 3:**
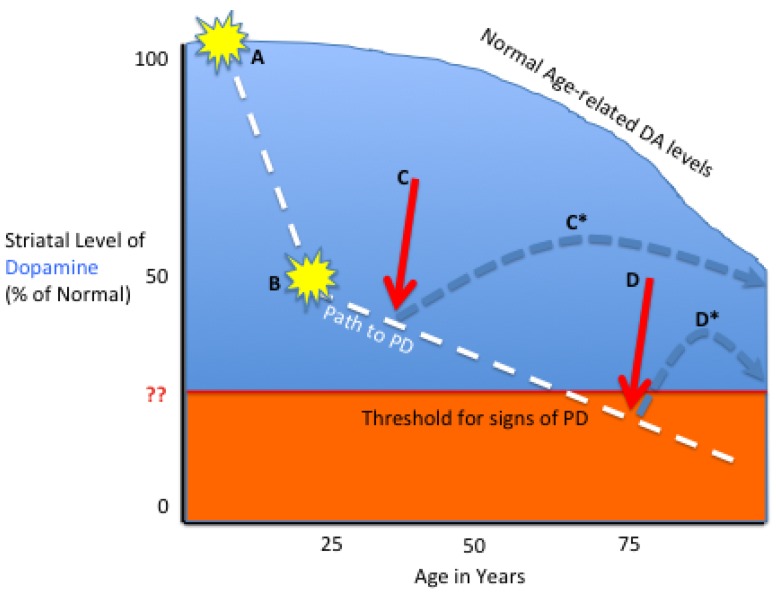
Schematic of Future Gene Therapy Options in PD. Normal aging is associated with gradual reduction in striatal DA, associated with reduced numbers of SNc dopaminergic neurons. Despite this age-related DA reduction, most individuals do not achieve a threshold level of dopamine depletion during life, and do not develop PD. In PD patients, however, hereditary (A) and/or environmental (B) factors/events lead to a more drastic DA depletion (Path to PD) that crosses the PD threshold and results in clinical signs. The slopes from A to B and in the Path to PD are variable. In cases of early PD, preferably before clinical signs are evident, GFL gene therapy (C) may protect from further nigrostriatal neuronal loss and restore near-normal DA levels (C*) for the patient’s life. In patients with symptomatic PD, use of AADC or AADC+ gene therapy (D), has the potential to increase striatal levels of dopamine above the threshold (D*) and restore more normal motor function.

The most lauded goals of gene therapy for PD are to address unmet medical need (Figure 3). In advanced patients with PD, whose pharmacological options have been exhausted, or have drug-resistant freezing of gait and marked fluctuations, it would appear that AADC gene transfer would hold promise for restoring levodopa responsiveness. In a similar manner, this may also be a patient population that would benefit from the tripartite gene reconstitution of the dopamine biosynthetic pathway. In early and midstage PD patients, who retain sufficient viable projecting nigrostriatal neurons, the combined neuroprotective and neurorestorative action of a GFL appears to have potential to modify the natural history of the disease. Finally, it is anticipated that safe and pharmacologically accessible gene regulation will be possible in PD gene therapy. This will offer the potential to “dial-in” therapeutic gene doses, as might be required to achieve clinical responses, as well as, to address safety issues that may attend constitutive production of transgene products.

### 3.2. Future Needs

A better understanding of the pathologic mechanism(s) responsible for PD in the majority of patients is greatly needed. Likewise, the ability to predict preclinical PD may allow earlier therapeutic interventions, especially those that can alter the course of the disease. With whole genome sequencing likely to redefine the landscape of PD, we anticipate that more individually targeted approaches will be heralded by new mechanistic data. The successful development and securing of a New Drug Application (NDA) for a single PD gene therapy from FDA will have greatly laid the groundwork required for moving other candidates forward. While still nascent as a therapeutic modality, CNS gene therapy is indicated to have a lasting and distinct clinical indication in the future.
